# Mitigating Coronavirus Induced Dysfunctional Immunity for At-Risk Populations in COVID-19: Trained Immunity, BCG and “New Old Friends”

**DOI:** 10.3389/fimmu.2020.02059

**Published:** 2020-09-04

**Authors:** Thomas-Oliver Kleen, Alicia A. Galdon, Andrew S. MacDonald, Angus G. Dalgleish

**Affiliations:** ^1^Immodulon Therapeutics Limited, Uxbridge, United Kingdom; ^2^Lydia Becker Institute of Immunology and Inflammation, Manchester Collaborative Centre for Inflammation Research, Faculty of Biology, Medicine and Health, Manchester Academic Health Science Centre, University of Manchester, Manchester, United Kingdom; ^3^Institute for Infection and Immunity, St George’s, University of London, London, United Kingdom

**Keywords:** COVID-19, SARS, dysfunctional immune response, vaccine, trained immunity, BCG, IMM-101, *Mycobacterium obuense*

## Abstract

The novel, highly contagious coronavirus SARS-CoV-2 spreads rapidly throughout the world, leading to a deadly pandemic of a predominantly respiratory illness called COVID-19. Safe and effective anti-SARS-CoV-2 vaccines are urgently needed. However, emerging immunological observations show hallmarks of significant immunopathological characteristics and dysfunctional immune responses in patients with COVID-19. Combined with existing knowledge about immune responses to other closely related and highly pathogenic coronaviruses, this could forebode significant challenges for vaccine development, including the risk of vaccine failure. Animal data from earlier coronavirus vaccine efforts indicate that elderly people, most at risk from severe COVID-19 disease, could be especially at risk from immunopathologic responses to novel coronavirus vaccines. Bacterial “new old friends” such as Bacille Calmette-Guérin (BCG) or *Mycobacterium obuense* have the ability to elevate basal systemic levels of type 1 cytokines and immune cells, correlating with increased protection against diverse and unrelated infectious agents, called “trained immunity.” Here we describe dysfunctional immune responses induced by coronaviruses, representing potentially difficult to overcome obstacles to safe, effective vaccine development for COVID-19, and outline how trained immunity could help protect high risk populations through immunomodulation with BCG and other “new old friends.”

## Introduction

In recent months, a novel severe acute respiratory syndrome (SARS) coronavirus (CoV), SARS-CoV-2, which causes COVID-19, has spread rapidly throughout the world (1). As of July 15, 2020, more than 13 million infections and over 575,000 COVID-19 related deaths have been confirmed worldwide. Based on a chronic lack of adequate testing capabilities in many countries worldwide, including large industrialized nations like the United States, a large amount of undiagnosed infection and mortality from COVID-19 must be assumed. The unprecedented pandemic seriously challenges the world’s health care systems and available hospital capacities to treat seriously ill patients. These challenges are amplified by frequent SARS-CoV-2 infection of healthcare workers (HCW), leading to hospital-acquired infection of HCW and patients, and significant mortality within that group (2). Other high-risk groups of infection include the elderly, with age-related immunosenescence and “inflammaging” having been suggested as a mechanism responsible for lowered immunological competence and the high mortality of the elderly in the current COVID-19 pandemic (3). Age-related risks are a particular issue in assisted care facilities and individuals with serious, non-COVID underlying health conditions like cardiovascular disease, chronic kidney disease, diabetes, chronic respiratory disease, immunosuppression, and cancer (4, 5). In the case of cancer, many malignancies require active treatment, making isolation – even social distancing – impossible, based on the need to commute to the hospital regularly to receive treatments. Therefore, there is an urgent need to protect individuals aged 55 years and older with co-morbidities. Throughout the public discourse, there has been little attention given to the observations that these populations are historically the same populations that are most unlikely to develop efficient and protective immune responses to standard respiratory viruses. Consequently, this is likely to be the same case for SARS-CoV-2. Indeed, for these populations, other more potent vaccines, compared to the general population, are required, e.g., “high dose” Influenza shots for the elderly. Nevertheless, those more potent vaccines often still result in less than ideal outcomes in these vulnerable populations (6). In order to avoid the need for achieving herd immunity by infection or mass vaccinations before safely reopening societies and economies, a priority would be immunizing the most at-risk populations first. There is a justified concern that suboptimal vaccine efficacy for at-risk populations and the elderly could place the goal of not having to achieve herd immunity first in jeopardy. At the same time, a non-efficacious vaccine for at-risk populations could increase the probability of second and subsequent waves of infection in these populations (7).

Worldwide availability of safe, effective, prophylactic vaccines is likely the only approach that will ultimately control this deadly pandemic. However, such vaccines may not be available until early next year, even in the most optimistic scenarios (8). Despite numerous efforts, no vaccine, proven safe and effective in humans, has ever been developed against any coronavirus (9, 10). Questions about the potential lack of sufficient vaccine efficacy in elderly populations have not yet been widely discussed. Therefore, strategies to prevent COVID-19 morbidity and mortality in high risk groups are desperately needed in order to safeguard the most vulnerable individuals, as well as maintaining continuous patient care and functioning hospital systems.

Both humans and animals are susceptible to disease caused by CoVs. Three highly pathogenic CoVs are known, SARS-CoV, Middle East respiratory syndrome (MERS)-CoV and SARS-CoV-2. All three are now known to efficiently infect and replicate in the lower respiratory tract, frequently causing substantial immunopathology, acute lung injury (ALI), acute respiratory distress syndrome (ARDS), and fatal pneumonia, resulting in high morbidity and mortality (11). SARS-CoV and SARS-CoV-2 are both members of the betacoronavirus genus and share more than 70% of their genetic code (12). However, it is noteworthy that SARS-CoV-2 is closest related to the bat coronavirus RaTG13, with 98% genetic similarity compared to all known genetic coronavirus sequences (13). Four additional, circulating but low pathogenic human coronaviruses (HCoV) are known and will not be reviewed here, but preexposure to them could impact the immune response to SARS-CoV-2 in patients (14). All four, HCoV-229E, HCoV-OC43, HCoV-NL63, and HCoV-HKU1, display a winter seasonality, causing comparatively mild to moderate upper respiratory illnesses and only occasionally, bronchiolitis and pneumonia symptoms (15, 16). All HCoVs share a minimum of four, genome encoded, major structural proteins: the spike (S) glycoprotein, nucleocapsid (N) protein, membrane protein (M), and the envelope protein (E), all of which are required to produce a structurally complete viral particle (17).

## Immune Responses to Coronaviruses

The pandemic, which originally emerged from Wuhan, China, has been characterized by a rapidly increasing morbidity and mortality rate associated with older age, beginning around age 50 years (18). Multiple aspects of immunity can be influenced by ageing, prompting scrutiny of which components of the immune response might be responsible for higher mortality in older people (19). In general, an early and robust innate immune response to viral infections permits more rapid and effective viral clearance and may even prevent symptomatic infection or diminish the severity of the infection (20). No correlates of protection have yet been formally established for the recently emerged SARS-CoV-2. However, mouse model data from studies with the first SARS-CoV that emerged in 2002, suggested a delayed innate immune response during infection is linked to a more severe course, with immunopathology in the lungs and high mortality (21). Initial observational studies suggest that a failure of antiviral immunity, including depleted natural killer (NK) cells, at an early stage in COVID-19, may lead to severe clinical course and an inability to recover from infection (22). In addition, it has previously been shown that the SARS-CoV macrodomain suppresses the innate immune response during infection, whereas an early strong innate immune response can protect mice from lethal disease and prevent detrimental downstream effects on the immune system (23). On the other hand, in later stages of infection, it appears that a dysregulated immune system, including excessive inflammatory responses by innate cells in the lungs, and selective immunosuppression of the adaptive immune system, can be detrimental for the host (24, 25). Acute lung injury caused by viruses like respiratory syncytial virus, influenza A virus and SARS-CoV have been described previously (11, 26, 27). Aberrant expression of the antiviral cytokine type I interferon (IFN), interferon stimulated genes, and other inflammatory cytokines, were observed in patients with severe SARS-CoV disease compared to healthy individuals, providing evidence that SARS-CoV is partly an innate dysregulated immune disease (28, 29).

The innate immune system recognizes pathogen-associated molecular patterns (PAMPs) of viral or bacterial intruders via pattern recognition receptors (PRRs). Toll-like receptors (TLRs), a family of type I transmembrane PRRs that consists of related, transmembrane proteins, play a central role in the initiation of inflammatory responses against pathogens, including the secretion of cytokines and chemokines. TLR4 is known to sense lipopolysaccharide (LPS) from gram-negative bacteria, but, based on its additional function as sensor for damage-associated molecular patterns (DAMPs), TLR4 has been suggested to play a central role in the induction of damaging inflammatory responses during several acute viral infections (30). In addition, oxidized phospholipids (OxPLs), DAMPs which lead to ALI in patients infected with SARS-CoV, also accumulate in lungs of patients infected with SARS-CoV-2 and activate monocyte-derived macrophages through TLR4 (31, 32). Interfering with innate cell activation by TLR4 in response to ligands such as OxPLs may therefore help prevent thrombotic complications, recently identified as a major factor in mortality of COVID-19 patients (33–35). Endothelial cell activation, infection and dysfunction has been implicated in severe COVID-19 by altering vessel barrier integrity, promoting a pro-coagulative state, inducing vascular inflammation, endotheliitis, and mediating inflammatory cell infiltration. The proposed mechanism is disruption of vascular integrity and endothelial cell death, which leads to exposure of the thrombogenic basement membrane and results in the activation of the clotting cascade (36). Altered platelet gene expression and functional responses in patients infected with SARS-CoV-2 may additionally contribute to observed hemostatic abnormalities like disseminated intravascular coagulopathy (37).

### Neutrophils

Neutrophils are an important component of the general response to infection in the respiratory system and capable of recognizing viruses via viral PAMPs (38). In the context of potentially excessive neutrophil activation in late stage COVID-19 disease, neutrophil extracellular traps (NETs) in the lungs can drive severe pathologies by accumulation of mucus in the airways of patients, contributing to ARDS (39). More importantly, NETs have been proposed to contribute to organ damage and mortality, since excess NET formation can trigger a cascade of inflammatory reactions that destroys surrounding tissues and facilitates atherosclerosis, aortic aneurysms, as well as thrombosis, including microthrombosis, in the vascular system, with devastating effects on organ function (39).

### Macrophages

Macrophages are key innate immune cells in any infection setting (40, 41). They are highly flexible innate cells that can, simplistically, be functionally and phenotypically divided into pro-inflammatory “M1” macrophages (capable producers of inflammatory cytokines and mediators, that kill infectious organisms, virus-infected cells, or tumor cells) and more regulatory “M2” macrophages (that are important for wound healing and parasite infections) (42, 43). Both activation states are needed for a “balanced” immune response, although the M1/M2 paradigm of macrophage activation is an over-simplistic definition of these complex and diverse innate cells (44, 45). During ageing and chronic inflammatory diseases, macrophages may switch to a more M2-like phenotype (46, 47). Importantly, nearly all identified high-risk factors for severe COVID-19 disease, like cardiovascular disease, diabetes, age, chronic obstructive pulmonary disease, and smoking, generally share a shift from more M1 to more M2 phenotype and function (48). Classical activation of M1 macrophages is induced by LPS/IFN-γ exposure, while alternately activated M2 macrophages are stimulated by IL-4, IL-10, IL-13 and glucocorticoids (49). The activation of innate immune cells such as macrophages can be heavily influenced by the character of the T cell response and, in particular, the cytokines produced by T cells during infection (50). SARS-CoV replication has previously been shown in human peripheral monocytes and macrophages, with varying efficacy. Importantly, the infection efficiency was shown to be donor dependent, with 100% infection in some and less than 5% in others (51).

### γδ-T Cells

In adults, Vγ9Vδ2 cells are the dominant γδ T cell population, however, in elderly individuals the variability increases (52, 53). An analysis of T cell repertoires in HCW who survived SARS-CoV infection during the 2003 outbreak revealed that an innate-like subpopulation of effector memory T cells, γδ-T cells, specifically Vγ9Vδ2 T cells, were selectively expanded approximately 3 months after the onset of disease (54). Importantly, no such expansion of non-innate αβ T cells was detected at the same time point. Furthermore, expansion of the Vγ9Vδ2 T cell population was associated with higher anti-CoV IgG titers, and *in vitro* experiments demonstrated that Vγ9Vδ2 T cells display an IFN-γ -dependent ability to directly kill CoV infected target cells. Therefore, innate-like Vγ9Vδ2 T cells may play a protective role during SARS-CoV and other CoV infections. A recent study analyzed the number and activation status of Vγ9Vδ2 T cells in hospitalized patients with COVID-19. They found significantly lower levels of Vγ9Vδ2 T cells than that of matched healthy control and concluded that this could indicate that elderly with lower frequencies of Vγ9Vδ2 T cells constitute a SARS-CoV-2 vulnerable population or that the Vγ9Vδ2 T cells in these patients have migrated to the lungs to kill SARS-CoV-2 infected cells (55).

### T Cells and NK Cells

SARS-CoV infection leads to lymphopenia and strongly reduced peripheral T cell levels, with low CD4+ and CD8+ T cell counts associated with adverse outcome, and a rapid and dramatic restoration of peripheral T cell subsets in the periphery of recovering patients (56–58). In addition, SARS-CoV can infect and replicate within PBMCs of SARS-CoV patients, with viral replication appearing to be self-limiting but leading to leukopenia or lymphopenia (59–61). Patients with clinical symptoms of severe COVID-19 also commonly present with lymphopenia, including dramatically reduced numbers of NK cells, CD4+ T cells, CD8+ T cells and B cells, which has not been observed in mild cases (62–65). Further studies have shown exhaustion markers like NKG2A on cytotoxic lymphocytes, including NK cells and CD8+ T cells, are upregulated in patients with COVID-19, and that for recovered patients, numbers of NK cells, CD4+ T cells, CD8+ T cells, and B cells normalize, along with markers of exhaustion on cytotoxic lymphocytes (66, 67). Reduced functional diversity and increased T cell exhaustion in peripheral blood could predict severe progression in COVID-19 patients, supporting the role of functional T cells in controlling COVID-19 (67). Importantly, it was recently shown that a patient with mild to moderate COVID-19 symptoms had a broad-based robust immune response across different immune cell types, which was associated with rapid recovery (68). This observational study identified the presence of activated CD4+ T cells, CD8+ T cells, and follicular helper T cells in the blood, along with increased antibody-secreting cells and IgM and IgG antibodies. The study did not investigate the neutralization capabilities of the observed antibodies. Cell-mediated type 1 immune responses are therefore theorized to be a major component necessary to overcome COVID-19 infection (69).

This is further supported by a study that screened for the presence of SARS-specific T cells in a cohort of three SARS-CoV-recovered individuals, where CD8+ T cell responses targeting the SARS-CoV membrane and nucleocapsid proteins were found to persist up to 11 years post-infection (70). Characterization of SARS-CoV-specific memory T cells from recovered individuals 4 years after infection indicated that the majority of memory CD8+ T cells produced IFN-γ, whereas memory CD4+ T cells produced IFN-γ, IL-2, or TNF-α (71). Multiple other independent studies established that SARS-CoV specific memory CD4+ and CD8+ T cells persisted for up to 2 years after infection (72–74). S protein-derived epitopes of SARS-CoV elicited recall CD8+ T cell secretion of IFN-γ as well as intracellular production of IFN-γ, TNF-α, perforin, and granzyme A from recovered patients over 1-year post infection, indicating that SARS-CoV infection can induce strong and long-lasting cytotoxic T lymphocyte (CTL)-mediated immunity in patients (75, 76). High frequencies of CD8+ Tc1-type T cells, reactive against MERS-CoV, were observed in a large proportion of patients with severe and moderate MERS at acute stage before detection of humoral and CD4+ T cell responses. Another report emphasizing the importance of T cells demonstrated that 17 years after the 2003 SARS outbreak, SARS-CoV-recovered patients still maintained long-lasting memory T cells reactive to the N protein of SARS-CoV, which notably exhibited robust cross-reactivity to the N protein of SARS-CoV-2 (77). A recent study showed predominant Th1 responses in convalescing COVID-19 cases, with little to no Th2 responses. It demonstrated SARS-CoV-2 specific CD4+ T cells in 100% of COVID-19 convalescent patients, with the majority of responses against S protein, correlating with the magnitude of anti-SARS-CoV-2 IgG and IgA titers, but as well responses against M and N proteins in all patients, accounting for 11–27% of the total CD4 + responses. The same study found SARS-CoV-2 specific CD8+ cells against S and M proteins in about 70% of patients, and interestingly, T cell reactivity to SARS-CoV-2 epitopes was also detected in non-exposed individuals, likely cross-reactive from previous, seasonal HCoV infections (78). However, at the convalescent phase, the magnitude of the CD8+ T cell response was not increased further.

Although it seems clear that robust inflammatory and CTL responses are required to clear the invading virus, when excessive, they can also lead to lung tissue destruction and pneumonia (79). Early pathological findings of COVID-19 patients with ARDS, showed not only reduced counts of peripheral CD4+ and CD8+ T cells, but that remaining T cells were found in a hyperactivated state, with high proportions of HLA-DR and CD38 double-positive fractions (80). It is noteworthy here that, in patients hospitalized with avian H7N9, survival reflected an early, but transient, prevalence of highly activated CD8+CD38+HLA-DR+PD-1+ T cells, but prolonged CD38+HLA-DR+PD1+ co-expression predicted fatal outcomes (81). CD8+ T cells in patients that died of H7N9 were non-functional, as reflected by a lack of IFNγ production, but displayed high and continued expression of the CD38+HLA-DR+ activation markers, together with the inhibitory PD-1 immune checkpoint receptor. Similar studies in Ebola, Dengue, and pandemic H1N1 have also mentioned the presence of these “non-survival” peripheral lymphocyte populations, with high and prolonged frequency of activated CD8+CD38+HLA-DR+ cells (82–84). We hypothesize that, as suggested for H7N9 disease (81), in COVID-19 patients this could also be associated with defective T cell activation and a lack of relevant T cell receptor (TCR) specificities. It is known that infection with human immunodeficiency virus (HIV) induces broad lymphocyte activation, with an increase in T cell activation markers such as CD38 (85). Several studies have shown that such increased CD38+ expression on CD8+ T cells is a strong predictive marker for disease progression in HIV-1 infection (86). Not only does the CD8+CD38+ T cell count predict progression of HIV disease to AIDS and death, but it is also independently predictive for evaluation of high plasma virus load and low CD4+ T cell counts (87). In early HIV infection, during onset of viremia, CD8+CD38+HLA-DR+ T cells correlate inversely with viral set point. However, hyperacute HIV infection leads these cells to be short-lived effector cells that do not persist, characterized by marked apoptosis, upregulation of CD95 and failure to upregulate the IL-7 receptor CD127 (88). Strikingly, in a recent study in COVID-19 patients, considerable proportions of peripheral CD4+ and CD8+ T cells co-expressed CD38 and HLA-DR, but those cells could not be re-activated with peptide pools of the S protein *in vitro*, supporting the notion of SARS-CoV-2 specific refractory T cells and/or different specificities (14). No data about the PD-1 status of T cells was provided. The same remarkable study showed that, while the majority of S-reactive CD4+ T cells from COVID-19 patients co-expressed CD38 and HLA-DR, S-reactive CD4+ T cells from healthy donors, proposed to be cross reactive to other HCoVs, only expressed CD38 and HLA-DR at very low frequencies and co-expression was not observed. In cancer therapy models, depleting “dysfunctional” CD8+CD38hiPD-1+ cells enhanced therapeutic outcomes, and patients who did not respond to immunotherapy showed more CD8+CD38hiPD-1+ in tumor and blood compared to responders (89). The potential significance of levels and timing of prolonged expression of CD38, HLA-DR, and PD-1 on dysregulated T cells and the utility of CD8+CD38+HLA-DR+PD-1+ T cells as a prognostic marker could be important and should be investigated in more detail. These could serve as indicators of SARS-CoV-2 immunosuppression, exhaustion and immune evasion, predicting divergent disease outcomes. The suggestion that a dysfunctional immune response is at the heart of COVID-19 pathology is further supported by the recent finding that, compared to patients with moderate disease, significantly reduced frequencies of CD8+ T cells, as well as diminished frequencies of CD4+ and CD8+ T cell subsets with activated differentiated memory/effector phenotype and migratory capacity, are found in peripheral circulation of patients with severe COVID-19 (90).

### B Cells and Antibodies

Antibody responses elicited by coronaviruses, including SARS, have been described as comparatively short lived and inconsistent (91, 92). Studies with human volunteers that were infected with a seasonal coronavirus HCoV-229E showed that individuals could get infected and display symptoms, including lymphocytopenia, regardless of preexisting antibodies (91). One study showed that six years post infection, SARS-CoV specific IgG was undetectable in 21 of 23 former patients, and no SARS-CoV specific memory B cell responses could be detected in any of the 23 patients (93). Another study revealed that SARS-CoV antibodies could be seen up to 24 months after infection (93). Interestingly, longevity of MERS-CoV antibody response correlated with disease severity. In one study, patients with severe MERS-associated pneumonia had a persistent antibody response detected for about 18 months after infection, while patients with infection limited to the upper respiratory tract or who had no clinical signs had no detectable MERS-CoV antibody response (94). In another report, the more severe the illness, the greater the antibody response, including IgM, IgG, and neutralizing Ab (NAbs). Patients in the convalescent phase, with mild or asymptomatic disease, rarely developed antibody responses (79). A strong antibody response developed in most MERS patients only after 2–3 weeks of illness, but the antibody responses were not correlated with the elimination of the virus from the body (95, 96). This was confirmed in two more studies that showed MERS infections are frequently characterized by low NAbs, despite patient recovery (93, 97–99). It is noteworthy that this was also recently shown for COVID-19 patients, where seroconversion has been observed in 9 mild to moderate cases after 6–12 days, but, despite COVID-19 antibodies arising at that time, no rapid decline of viral loads was observed, as would be expected with highly effective and neutralizing antibodies (100). Since anti-SARS-CoV antibody responses are short-lived in patients who have recovered from SARS, there are early indications that antibodies, and especially NAbs, may not be the predominant mechanism necessary for effective viral clearance and for infected individuals to overcome a COVID-19 infection (10, 101–103). This is further reinforced by the first longitudinal study in COVID-19 patients, which showed that some individuals who have recovered and displayed a strong NAb response shortly after infection, had titers fall as much as 23-fold, and in some cases back to baseline within 3 months (104). The authors speculated that the observed transient NAb response could be a feature shared by both a SARS-CoV-2 infection that causes low disease severity, and the circulating seasonal coronaviruses. Other recent data supports the notion of an unclear role of Abs, by reporting short duration of Ab and NAb titers after SARS-CoV-2 infection. Compared with responses of patients with symptoms, asymptomatic individuals (arguably with the more effective immune response), had weaker Ab responses to infection, with a reduction of IgG levels already occurring in the early convalescent phase (105); viral load and duration of infection are likely to be factors. Remarkably, in this study, 40% of asymptomatic patients had undetectable levels of protective antibodies two to three months after infection, compared to 13% of the symptomatic patients with COVID-19. An even more notable finding, further indicating a limited role for Abs in overcoming SARS-CoV-2 infection, is that intrafamilial exposure to SARS-CoV-2 induces a cellular immune response without seroconversion (106).

### SARS Vaccine Challenges

Of the different proteins that characterize coronaviruses, the S protein is an important determinant of virulence, tissue tropism and host range (107). Trimers of S form the characteristic large spikes on the coronavirus envelope and both SARS-CoV and SARS-CoV-2 use the protein angiotensin converting enzyme 2 (ACE-2) as primary receptor for docking and infecting human host cells. Priming of the virus S protein by host cell proteases is essential for entry. When SARS-CoV-2 docks to the cell via the ACE-2 receptor, the host transmembrane serine protease 2 (TMPRSS2) is responsible for cell entry (108–110). TMPRSS2 also aids the MERS-CoV to penetrate the cell (111), but its primary receptor for entry is dipeptidyl peptidase 4 (DPP4) (112). Virus S glycoproteins are postulated to elicit an immune response in humans that could protect against future infection (108, 113). Many vaccine approaches against COVID-19 that are currently in development are focusing primarily on the generation of antibody responses against the SARS-CoV-2 S protein (8). However, despite the great urgency for making an effective vaccine against COVID-19 available, this approach must be undertaken with great caution. Several SARS-CoV vaccines that initially induced antibodies and short-term protection in mouse models of SARS-CoV led to dysfunctional or type 2 helper T cell (Th2)-type immunopathology on challenge, with prominent eosinophil infiltration in the lungs, suggesting hypersensitivity to SARS-CoV components was induced (10). Several other independent studies with animal models used to develop vaccine candidates against SARS-CoV exposed signs of lethal vaccine failure based on induction of cell-mediated type 2 enhanced immunopathology, with associated eosinophilic infiltrates causing severe pneumonia, especially in aged mice. A vaccine based on SARS-CoV S protein protected against viral challenge when young mice were vaccinated, but it failed to efficiently protect older mice (114). Another study indicated poor vaccine performance as well as Th2-based eosinophilic immune pathology in the lungs that was shown to be caused by alum adjuvanted and unadjuvanted SARS-CoV vaccines in aged animals (115). All this requires that particular attention be given to the strongly increased mortality rate already evident in older SARS-CoV-2 patients and patients with comorbidities. SARS-CoV has been shown to dysregulate the immune response in SARS patients by biased activation of a Th2 response, which can counter-regulate the type 1 response that normally attacks bacteria and viruses (97). There was a significant increase in Th2 cytokines IL-4, IL-5 and IL-10 during acute infection in fatal SARS cases, once again indicating that the character of cellular immune response induced by any COVID-19 vaccine will be critical in determining whether it will succeed (101, 116). Four earlier vaccines against MERS-CoV-2 have been tested in rhesus macaques (RM), but no reports of efficacy of a single-dose MERS-CoV vaccine in non-human primates (NHPs) had been made until a recent study reported that RM seroconverted after a single intramuscular vaccination with the experimental ChAdOx1 MERS vaccine (117). The study showed that vaccinated animals developed a neutralizing antibody response, were protected against respiratory injury and pneumonia, and showed reduced viral load in lung tissue and reduced disease severity. In addition, a Phase 1 trial in healthy individuals aged 18–50 years has been conducted, with no adverse safety signal reported (118). Neither study has provided data in either aged animals or elderly humans. Most relevant in this context are early SARS-CoV-2 vaccine trial data. A Phase 1/2 study in adults aged 18 to 55 years of a COVID-19 RNA vaccine candidate (BNT162b1), utilizing mRNA that encodes trimerized SARS-CoV-2 spike glycoprotein, showed the generation of NAb titers 28 days after the first injection and one week after the second dose (119). It is not yet known what kind of immune response the vaccine will elicit in older people or long-term. An additional mRNA nano-particle based vaccine candidate (mRNA-1273) has been reported to induce both potent Nabs and CD8+ T cell responses and to protect against SARS-CoV-2 infection in the lungs and noses of a mouse model, without evidence of immunopathology (120). Importantly, it showed spike peptide-reactive CD4+ and CD8+ T cells producing IFN-γ, IL-2, and TNF, which would be encouraging if corroborated in ongoing Phase 2 clinical trials and Phase 3 efficacy evaluation of the same vaccine candidate. Another advanced SARS-CoV-2 vaccine candidate in Phase 1 clinical studies is adenovirus-vectored vaccine ChAdOx1 nCoV-19, which has been reported to prevent SARS-CoV-2 pneumonia in RM and not to be Th2 dominated, determined by IgG subclass and cytokine expression profiling (121). Notably, no evidence of immune-enhanced disease following viral challenge twenty-eight days after vaccination was observed in the respective animals. The levels of Abs produced by the vaccine in these RM were lower than many Ab responses in humans infected with SARS-CoV-2. While the vaccine protected RM from severe infection, they became infected with evident active virus replication, which does not rule out the potential of maintained ability to transmit virus.

Despite the inherent challenges of adopting new routes of routine vaccine administration during an ongoing pandemic, recent evidence would encourage consideration of intranasal administration, inhalation or other vaccine strategies that directly target the mucosal surfaces of the airways, because of distinct functional responses by respective tissue-resident memory T cells (122). It was shown, for example, in a mouse model, that conserved epitopes shared by SARS-CoV and MERS-CoV could induce airway memory CD4+ T cells producing IFN-γ which were phenotypically and functionally different from lung-derived cells and crucial for protection against both CoVs. It is particularly noteworthy in this study that intranasal (but not subcutaneous) vaccination protected mice from pathogenic human CoVs, and that protection required IFN-γ and was depended on early induction of robust innate and virus-specific CD8+ T cells (123).

SARS-CoV-2 has shown replication, not only in human peripheral monocytes and macrophages, but also to directly infect T lymphocytes during primary infection through S protein-mediated membrane fusion, likely contributing to the severe lymphocytopenia that is a diagnostic indicator common in COVID-19 patients (51, 100, 124). SARS-CoV has also been shown to infect dendritic cells (DC), the central coordinators of the immune response, leading to impaired DC maturation and their high expression of the pro-apoptotic protein TRAIL (125). Instead of facilitating lymphocyte activation and expansion in numbers, this likely induces lymphocyte death and represents another mechanism of immune escape and intensification of the immunocompromised state of SARS-CoV patients (126). Similar mechanisms could contribute to lymphopenia and dysfunctional immune responses observed in severe COVID-19 patients. In the elderly, immune evasion by SARS-CoV-2 is probably made worse due to the reduced number and function of antigen presenting cells (APCs) (127). Multiple studies have been performed in mouse models describing the importance of type 1 CD4+ and CD8+ T cells in SARS-CoV (128, 129), with one study establishing that virus-specific memory CD8+ T cells provided substantial protection from lethal closely related SARS-CoV infection in a mouse model, emphasizing the importance of a cell-based type 1 immune response for survival of SARS infections (102). The majority of the many current vaccine strategies against SARS-CoV-2 rely on unadjuvanted or self-adjuvanted vaccines (e.g., RNA and DNA vaccines), or type 2 immune response promoting vaccines (e.g., alum adjuvanted, or unadjuvanted peptide or protein based vaccines) (113, 130, 131). Rather than promoting type 1 immunity, such approaches are likely to mostly lead to induction of type 2 responses which, as previously discussed, are unlikely to be effective against SARS-CoV-2 (100). Existing CoV antibodies have, in the case of host challenge with the same virus, enhanced viral load and disease severity in feline coronavirus or feline infectious peritonitis virus (FIPV) infections. This phenomenon is known as antibody-dependent enhancement (ADE) of viral infection (132, 133). In FIPV infection ADE can be induced by the presence of sub-neutralizing levels of anti-FIPV spike antibodies (134). Unlike in dengue virus infections, ADE in feline coronavirus infection is caused by re-infection with the identical serotype virus (124). It should be noted that mice, often used for preclinical safety evaluation of vaccines, lack FcγRIIa, the main FcγR on human cells linked to ADE induction (135, 136). Increasing viral entry into permissive cells and/or triggering excessive production of pro-inflammatory cytokines has made ADE a significant concern with several viruses, including the closely related SARS-CoV (137, 138). Concerns have also been raised that anti-SARS-CoV-2 non-neutralizing antibodies, or even declining NAb titers over time, could lead to ADE and enhanced disease after such vaccinations, antibody-based drug therapies, or treatment with convalescent plasma from recovered patients (139, 140). However, none of the early clinical trial results of the most advanced vaccine candidates described above have reported signs of ADE (121). Demonstration of a lack of ADE induction of different experimental vaccines against SARS-CoV-2 in NHPs and humans will remain critical for other vaccines advancing through the pipeline. One recent example of the need for continued vigilance is a study using Chinese macaques indicating cause for concern by showing that vaccine-induced, S-specific immunity in the form of anti-spike IgG resulted in severe ALI by skewing macrophage responses during subsequent, acute infection with closely related SARS-CoV (139).

Given all of the above, it is likely that successful vaccines against COVID-19 will require appropriate DC activation, leading to induction of a multifaceted and long-lived type 1 immune response that includes memory CD4+ Th1 cells, CD8+ CTLs, and NAbs. Most importantly, they will need to be effectively induced and sustained in older individuals without generating type 2 responses or ADE. It may remain a challenge to achieve this formidable goal and more creative approaches to vaccination may be required, but early data from pre-clinical and clinical trials of SARS-CoV-2 vaccines seem encouraging that they will provide some protection.

### Cytokine Storm

Direct comparisons in the literature of clinical observations in COVID-19 patients with IL-6 induced “cytokine storm” or cytokine release syndrome (CRS) should be made with caution (25, 141, 142). For example, cytokine levels during hyperinflammation in COVID-19 are multiple orders of magnitude lower than has been observed during cancer treatments by adoptive cell transfer of autologous T cells modified with chimeric antigen receptors (CAR-T cell therapy), a classical example for CRS (143, 144). Although, CRS is normally treated with extensive use of steroids, the clinical evidence does not support corticosteroid treatment for COVID-19 induced lung injury and interfered with clearance (145). In SARS and MERS, corticosteroid use did not improve patient mortality and also resulted in delayed viral clearance (11). It should be noted that a recent preprint of a randomized-controlled trial observed that therapy with dexamethasone lead to a significant reduction of death in ventilated patients, as well as for patients on supplemental oxygen, while no benefit was shown in mild cases (146). A recent review of corticosteroid use in the management of COVID-19 revealed a mixed picture from five available studies. In four retrospective studies and one quasi-prospective study, three studies indicated a benefit, while the other two studies showed no benefit, and one sub-study even suggested significant harm in critical cases (147). Based on success in hematological and oncology settings, several IL-6 antagonists (tocilizumab, sarilumab as well as siltuximab) have been utilized as emergency interventions in COVID-19 patients with ARDS and hypotension, although so far with mixed results (148). IL-6 is an indispensable cytokine that initiates innate defence after pathogen invasion or tissue damage by stimulating acute phase reactions, immune responses, hematopoiesis, and activation of numerous internal organs to prepare for host defence (149). Therefore, IL-6 and other cytokines like tumor necrosis factor (TNF)-α are indispensable during functional activation of monocytes, macrophages and DCs before or early during COVID-19 disease, as they are in diseases caused by other respiratory viruses (150). However, in later disease stages increasing immune dysregulation and T cell apoptosis, macrophages and IL-6 may accelerate immune imbalance (151).

Preventing and treating coronavirus infections will likely need a multiphasic approach to prophylaxis and therapy, especially in vulnerable populations. It will be important to use the right tools at the right time to avoid unintended and potentially counterproductive consequences. The right set of immunomodulators would likely be able to prepare and boost innate immune defences to either ensure appropriate, effective responses to infection and/or guide the development of suitable, protective immunity in response to potentially suboptimal adjuvanted first generation vaccines. Antiviral treatments or combinations of them will be most useful during early infection, while a different set of immunomodulators may be needed in late stage and severe disease, where a dysregulated antiviral response can cause deadly collateral damage.

## Microbial “Old Friends” and BCG

Some microbes have existed throughout human history, with evidence of their presence in hunter-gatherer societies, shaping the evolution of the human immune system (152). Some of these microbes, branded as “old friends” or “old infections,” are thought to be so intricately involved in this process that they are required for human immunity to develop and function properly (153, 154). Examples of such microbes are harmless mycobacteria that are present in the environment and used to be prevalent in water and food, where they were postulated to have a “training” impact on the human immune system (153). In addition, “paleolithic” strains of *Mycobacterium tuberculosis* (Mtb) that were less pathogenic than modern strains could have contributed to this process (152). Environmental Mycobacteria can provoke type 1 responses, as has been shown in mouse models and human cell-based *in vitro* studies for heat killed *Mycobacterium obuense*, NCTC13365 (IMM-101) and *Mycobacterium vaccae*, NCTC11659 (IMM-201) (155–158). This is also the case for the attenuated strain of *Mycobacterium bovis*, BCG (159). However, modern, urban societies are often missing frequent exposure to environmental bacteria such as *M. obuense* and *M. vaccae* – they literally have lost touch with their “old friends” and may need “new old friends,” to support type 1 immune responses.

Remarkably, several observational studies have recently proposed that countries with active BCG vaccination in place had fewer confirmed COVID-19 cases and related deaths (160–162). These observational studies should be appraised with caution, since there are many confounding factors in interpreting such correlative data in the context during the COVID-19 pandemic (163). There is no peer-reviewed data yet, or a clear scientific hypothesis about the proposed mechanism of action, to explain how decades later a single BCG vaccination could provide long lasting, heterologous protection against a viral disease. In contrast, there are evidence-based arguments, acutely relevant to the COVD-19 pandemic, regarding how BCG or type 1 immune inducing environmental Mycobacteria could provide protection against severe COVID-19 in the form of the trained immunity hypothesis.

## Trained Immunity Utility for Vaccines

Contact with specific microbial stimuli can induce long-lasting epigenetic changes in innate immune cells, which not only results in an enhanced response to a second challenge by the same microbe, but also to unrelated microbial insults (164). Referred to as “trained” immunity or innate immune memory, this process was originally shown for the BCG vaccine (165, 166). This concept may help explain previous observations that, after infection or vaccination, prototypical innate immune cells like monocytes, macrophages and NK cells undergo long-term changes in their functional programs, promoting host resistance against a wide spectrum of pathogens, including fungi, bacteria and viruses (167). Trained immunity is thought to be responsible for the observation in clinical studies that childhood vaccination with BCG correlates with protection against 30–50% of infections with any known pathogen, including viruses (168, 169). Additionally, a reduction in childhood mortality, unrelated to the prevention of tuberculosis (TB), has been observed (169). Similar positive effects have been shown for BCG vaccinations in adults, including improving responses to Influenza vaccination (170). A study in Guinea-Bissau showed that BCG reduced the incidence of respiratory syncytial virus infection (171). Importantly for the at-risk populations for severe COVID-19, it was shown that BCG had a similar protective effect on respiratory tract infections in older individuals in Indonesia (172). In addition, a clinical trial performed in older individuals in Japan established protection against pneumonia after pneumococcal, influenza and BCG vaccinations (173). Further confirmation of this effect has been demonstrated in a randomized controlled trial in which BCG vaccination protected against experimental infection of a yellow fever virus (174). In summary, BCG vaccination has been shown to protect against a range of viral infections (175). Related to this, when vaccination against smallpox was introduced around 200 years ago, positive side-effects such as protection against measles, scarlet fever and whooping cough, among others, were noticed (176).

Monocytes from healthy human volunteers were stimulated *ex vivo* with unrelated pathogens and displayed enhanced pro-inflammatory cytokine production of IL-1β, TNF and IL-6 after BCG vaccination (165). Experimental studies in mice have delineated that some of the mechanisms by which BCG induces these protective effects. For example, in mice, reduced viral titers of influenza A virus rely on macrophages (177). Subcutaneous administration in mice of muramyl dipeptide (MDP), part of the mycobacterial cell wall, protected against vaccinia virus and herpes simplex virus type 2 (HSV2) infections (178). Newborn mice could be protected with BCG from infection by HSV2 (179). More recently, other inducers of trained immunity have also been identified, including β-glucan, which has been shown to induce protective trained immunity in human monocytes and against Mtb infection in mice (180). The combination of these observations and others led to the proposal of the development of trained immunity-based vaccines (TIbV).

TIbVs aim to induce a pre-activated or “poised” activation state in innate immune cells. In this way they are, unlike conventional vaccines, theoretically able to stimulate much broader immune responses that are not focused on just one specific pathogen (181). This capacity of TIbVs to promote responses beyond their nominal antigens may be particularly useful when conventional vaccines are not available, or when multiple co-infections and/or recurrent infections arise in susceptible individuals at the same time, as is the case in the current pandemic COVID-19 health emergency. At least six different countries, including the Netherlands and Australia, have initiated clinical trials with the intent of investigating BCG vaccination as TIbV to protect HCW from symptomatic or serious COVID-19 infections (175, 182, 183).

In general, BCG is regarded a safe vaccine in young and healthy individuals. However, as is the case with any vaccines containing live attenuated organisms, there is a possibility of adverse events, such as disseminated BCG disease, in the elderly and immunocompromised. For this reason, in cancer patients, who represent a high-risk group for severe COVID-19 infection, BCG is contraindicated in several countries highly impacted by the pandemic, including the United States and Canada (184, 185). As a result, populations likely to benefit most from the potential of TIbVs and at the highest risk of a severe COVID-19 disease (e.g., cancer patients, frail elderly, or other people with impaired immune systems), cannot be included in BCG vaccination strategies. Despite the potential promise for mitigation of the COVID-19 pandemic, a major obstacle to its quick, rational deployment is the fact that the BCG vaccine comprises of a number of genetically distinct substrains (186). These have subsequently been shown to have different immunological properties, such as variable virulence and efficacy as a tuberculosis vaccine in mice (187). This substrain diversity may also help explain some inconsistencies following BCG use, such as variable Th1 or Th2 induction and side-effects (188). In clinical use, no evidence was found that vaccination efficacy against TB was associated with a specific BCG strain; however, a Th1 or Th2 bias was not investigated in that study (189). It has also been shown that the immune response can be directed from Th1 to mixed Th1/Th2, depending on the dose of BCG used (190). Bacille Calmette-Guérin is not routinely injected more than once, but an earlier study showed that, of six patients who were given a second inoculation of the BCG vaccine, three showed persistent cutaneous granulomas (191). A recent clinical study also observed evidence of a protective effect against persistent Mtb infection after BCG revaccination (192); although repeat treatment with BCG has been used in the past in oncology as an adjuvant to boost cell-based cancer vaccines (193).

### *Mycobacterium obuense* (IMM-101)

**FIGURE 1:**
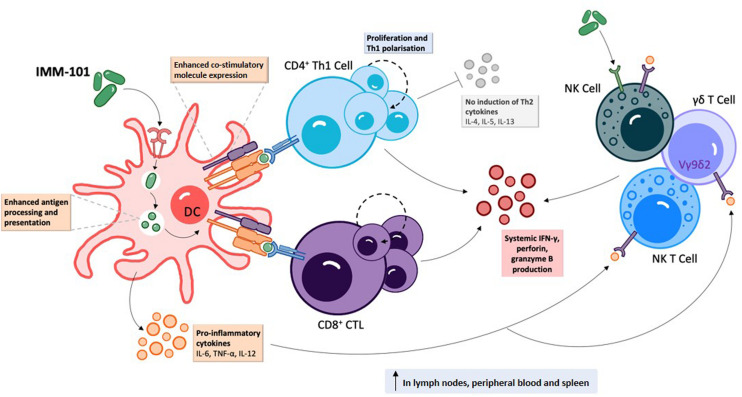
IMM-101 induces a robust systemic type-1 biased immune response | Recognition of IMM-101 by DCs results in increased expression of co-stimulatory molecules, enhanced antigen processing and presentation capacity and induction of an array of pro-inflammatory cytokines 156, 158, 198–202). IMM-101 activated dendritic cells (DC) directly promote the proliferation of CD8+ cytotoxic T-lymphocytes (CTL) and type-1 polarised CD4+ T cells, whereas innate-like cells including natural killer (NK), NKT and γδ T cells can be activated either by direct interaction with IMM-101 or indirectly via recognition of DC secreted cytokines (156, 157). This local DC activation eventually leads to a systemic increase in immune cells secreting anti-viral interferon (IFN)-γ, perforin and granzyme B (158, 202). Th, helper T cell. TNF, tumour necrosis factor.

**FIGURE 2:**
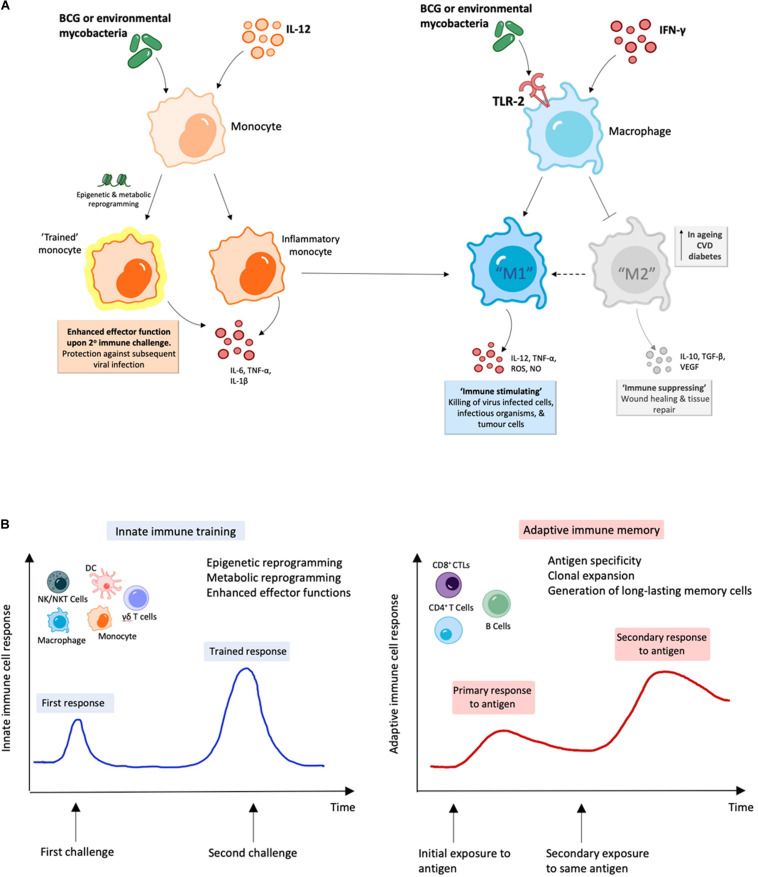
BCG and environmental mycobacteria promote M1 macrophages and are likely to induce trained immunity. **(A)** Treatment with mycobacterial immunomodulators induce polarization of M1 macrophages along with “trained” inflammatory monocytes with enhanced M1 function, which can result in enhanced viral clearance (164–166, 199). **(B)** During innate immune training, innate cells undergo long-term cellular reprogramming. Unlike classical antigen-specific responses seen with adaptive immunity, this reprogramming results in increased capacity to respond to secondary challenges from a variety of pathogens and forms the basis of trained-immunity based vaccines (170–172, 181).

IMM-101 is a preparation of heat killed, whole cell, *M. obuense* National Collection of Type Cultures (NCTC) 13365, one of over 100 named species within the genus *Mycobacterium*, and an “old friend.” *M. obuense* is a rapidly dividing mycobacterium that normally grows as an environmental saprophyte (194). Since IMM-101 is a heat killed preparation, treatment is not associated with the potential side-effects of delivering live or attenuated organisms (195). Moreover, one can speculate that IMM-101, by virtue of its potent type 1 inducing ability, will counter-regulate type 2 responses, helping to explain the encouraging clinical results to date in melanoma and pancreatic cancer (195, 196). An open label, Phase 2 study of IMM-101 in combination with checkpoint inhibitor therapy Nivolumab is currently underway in patients with advanced melanoma in the United Kingdom (197). The total number of patients exposed to IMM-101 across clinical trials and compassionate programs without any unexpected adverse events has been over 345. The mode of action of IMM-101 is in the process of being elucidated, but it has been shown to be a multifaceted modulator of both innate and adaptive arms of the immune system (158). Experiments with mouse and human immune cells have shown that IMM-101 is very effective in inducing cytokine expression by innate immune cells, including M1 polarization and enhanced antigen presentation by DCs, leading to a typical type 1-biased immune response (Figures 1, 2) (198, 199). Systemic activation of, and IFN-γ production by, multiple immune cell types (158), including innate immune cells like NK cells, T cells expressing gamma/delta receptors (γδ-T cells) and natural killer T (NKT) cells (157, 200) (Figure 1), is based in part on the promotion and activation of CD4+ Th1, and CD8+ CTLs, with increased production of the cytokine IFN-γ in *in vitro* and *in vivo* (198–202). It is also possible that, in this setting, IMM-101 may act to train monocytes for enhanced M1 function (Figure 2). NK, γδ-T, NKT, Th1 cells, and CTLs, are well-known to play crucial roles in anti-viral and anti-tumor responses that can kill infected or tumor cells. This diverse mechanism of action of IMM-101, the safe promotion of a broad, systemic innate and adaptive type 1 immune response, may provide a rationale for considering its use against SARS-CoV-2.

Interestingly, BCG has been shown to promote activation of Vγ9Vδ2 T cells, the major subset of γδ T cell pool in human peripheral blood with a previously proposed protective role against SARS-CoV (see above) (203). Vδ2 T cells are exactly the cell-subtype that has been shown to also be activated by IMM-101 stimulation, in some experiments showing a stronger ability to do so than BCG (157). γδ T cells normally only represent a minor subset in peripheral blood, but can rapidly proliferate following infection with certain pathogens, expanding from 1% to over 50% of circulating T cells within a week (204, 205). It is noteworthy that a large majority of Vδ2 T cells co-express Vγ9 in humans, and were shown to be important to overcome SARS-CoV infection (54, 206).

In addition to Th1 cells, CTLs and γδ T cells, NK and NKT cells also play key protective roles during viral infection (207, 208), and the potential importance of improving the NK cell and CTL response at the early stage of SARS-CoV-2 infection has already been highlighted (22). Under the umbrella of trained immunity, broad protection could be achieved by systemically increasing the non-specific effector response of innate immune cells (e.g., macrophages, NK, NKT, and γδ T cells) while also enhancing DC activation and ability to promote adaptive T cell (e.g. Th1 and CTL) and B cell responses to both specific and non-related (bystander) antigens, all of which have been shown for IMM-101 (Figure 1) (198, 199).

Several studies have shown that the effects of IMM-101 are in part mediated by TLR2/1, and to a lesser extent, TLR2/6 (198, 199). TLR2 has been shown to directly trigger Th1 effector functions in mice (209). Subsequently, it was shown that IMM-101 activates human Mincle reporter cell lines (158, 227). It is noteworthy that Mincle can suppress TLR 4 activation (211) and TLR4 has been proposed to have a central role in the initiation of damaging inflammatory responses during different acute viral infections (30). In contrast to BCG, IMM-101 does not activate TLR4 (198, 199, 212). In a similar manner, Mincle suppresses Th17 immune responses, which as well have been suggested in coronavirus immunopathology and vaccine-induced immune enhancement (213, 214). It was only recently discovered that activation of the Mincle receptor is a key activation pathway for Complete Freund’s Adjuvant (CFA), the “gold standard” adjuvant for eliciting cell-mediated immunity (CMI) in research models (215–217).

Effective and enhanced viral and tumor antigen cross-presentation requires TLR2 or TLR3 activation of human DCs (218). Mouse CD8α+ DCs express TLR7 and TLR9, in addition to the TLR2 family and TLR3, whereas the only relevant corresponding cross-presenting human CD141+ DCs in lymph nodes exclusively express the TLR2 family and TLR3 (218, 219). Importantly, analysis of the susceptibility of primary human DC subsets to viral infections has shown that CD141+ DCs have an innate resistance to infection by a broad range of enveloped viruses, including HIV and influenza virus. In contrast, CD1c+ DCs are susceptible to infection, which enables viral antigen production, but impairs their immune function and survival. This has led to the conclusion that inclusion of TLR2 or TLR3 agonists would be the most direct mechanism to enable enhanced viral and tumor antigen cross-presentation, likely necessary for effective cancer immunotherapy (218) and viral clearance (220). Interestingly, previous work has suggested that vaccine-induced eosinophil immunopathology in the lungs after SARS-CoV infection could be avoided with the use of TLR3 agonists as adjuvants (221). However, use of TLR3 agonists may have to be viewed with caution in the context of COVID-19, based on observations of harmful contributions of TLR3 to influenza A virus-induced acute pneumonia in mice. In that scenario, TLR3-influenza A virus interaction critically contributed to the debilitating effects of a detrimental host inflammatory response (222). Further, it has been shown that TLR4 signaling induces TLR3 up-regulation in alveolar macrophages during ALI, and that TLR4 and TLR3 in macrophages are an important determinant in ALI (223), and that there is an association between respiratory syncytial virus TLR3-mediated immune responses and chronic obstructive pulmonary disease exacerbation frequency (224). TLR2 activation of macrophages leads to M1 polarization, and a shift from M2 into M1 macrophages (225). In addition, it has been shown that TLR2 activation of macrophages can impair activity of M2-like macrophages (226). IMM-101 activates TLR2 and not TLR4 and leads to M1 macrophage polarization (Figure 2) (198, 199). The combined characteristics of IMM-101 have led to the approval by Health Canada of a randomized, Phase 3 trial of immunization with IMM-101, versus observation, for the prevention of severe respiratory and COVID-19 related infections in cancer patients at increased risk of exposure (210).

## Discussion

**FIGURE 3:**
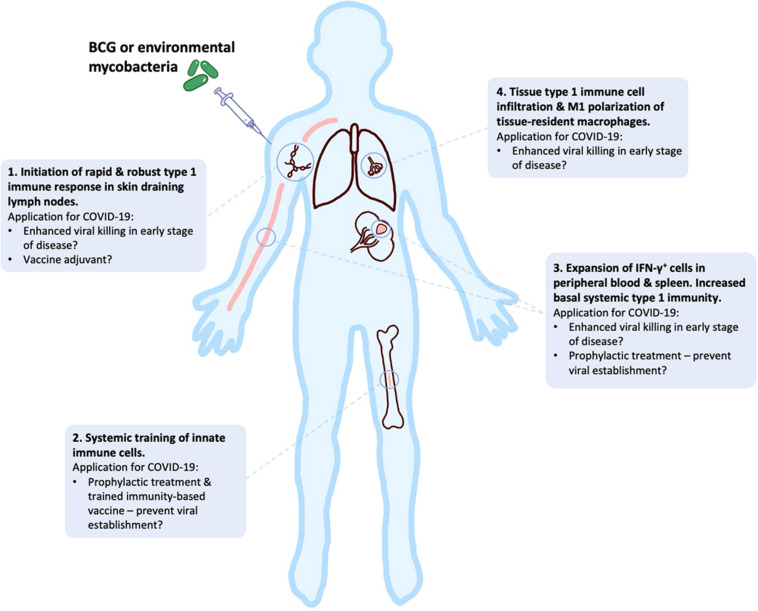
BCG and “new old friends” have potential utility for prevention of severe COVID-19 in a number of ways. Bacille Calmette-Guérin (BCG) and other mycobacterial immunomodulators initiate robust type 1 immune responses and innate immune training, leading to tissue type 1 immune cell infiltration and elevated basal systemic type 1 inflammation (156–159, 164–166, 198–202). This allows for potential alteration of disease trajectory through prevention of viral establishment, enhanced viral killing or as a vaccine adjuvant to enhance immunity.

In this review, we have presented an overview of current knowledge of the innate, adaptive and dysfunctional immune responses to SARS-CoV-2, in relation to other closely related coronaviruses. We have outlined the responses that may be required for successful vaccine development against COVID-19, while highlighting potential risks during this development, especially for the elderly. Early clinical data look promising, but continued studies of human and NHP immune response to different SARS-CoV-2 vaccines in the pipeline are required to mitigate potential dangers of well-intended, but potentially flawed, vaccines that are being expedited to large parts of high-risk populations around the globe. In addition, the potential utility of “new old friends” as TIbVs like BCG or heat killed environmental bacteria such as IMM-101, that act as multitargeted, systemic immunomodulators of the innate and adaptive immune system have been described. Studies to show BCG’s and IMM-101’s potential utility for the prevention of severe COVID-19 are underway or planned, with the potential to change immune status and alter disease trajectory in multiple ways (Figure 3): (i) as prophylaxis, with enhanced innate memory and increased basal systemic type 1 immunity preventing viral establishment; (ii) as a treatment for patients in early stages of disease, with increased local and systemic type 1 inflammation enhancing killing of virally infected host cells; (iii) as an adjuvant for future COVID-19 vaccines. Thus, BCG and IMM-101 have the potential to be rapidly deployed to address the COVID-19 emergency and the challenge posed by the current lack of effective treatments and vaccines, leading to a high unmet medical need. With other routes of vaccine and therapy development likely to take many months or years to develop, or even reformulate, the help of “new old friends” such as BCG and IMM-101 may be precisely what we need in the current pandemic crisis.

## Author Contributions

T-OK and AD conceived the idea for the review and wrote the manuscript, with constructive input from AM and AG. AG prepared display items under the supervision of AM. All authors approved the final version of the manuscript.

## Conflict of Interest

T-OK is an employee of Immodulon Therapeutics Ltd. AM is a member of the Scientific Advisory Board for Immodulon Therapeutics Ltd. AG is a recipient of an MRC (UK) NPIF PhD studentship, which is part funded by Immodulon Therapeutics Ltd. AD is a member of the Scientific Advisory Board for Immodulon Therapeutics Ltd.
